# Viscoelastic properties of dystrophin‐deficient mouse skeletal muscles are resilient to isometric fatiguing exercise

**DOI:** 10.14814/phy2.70841

**Published:** 2026-03-19

**Authors:** Deirdre L. Merry, Pavithran Devananthan, Natalia Kabaliuk, Angus Lindsay

**Affiliations:** ^1^ School of Biological Sciences University of Canterbury Christchurch New Zealand; ^2^ Biomolecular Interaction Centre University of Canterbury Christchurch New Zealand; ^3^ Department of Mechanical Engineering University of Canterbury Christchurch New Zealand; ^4^ Department of Medicine University of Otago Christchurch New Zealand; ^5^ Maurice Wilkins Centre for Molecular Biodiscovery Auckland New Zealand

**Keywords:** Duchenne muscular dystrophy, exercise, muscle stiffness, rheology, viscoelasticity

## Abstract

Exercise prescription for Duchenne muscular dystrophy (DMD) is complicated by the susceptibility of unstable skeletal muscle to contraction‐induced damage. Although evidence suggests that isometric contractions can confer molecular and physiological benefits to DMD muscle, their impact on viscoelastic properties has not been assessed in vivo. Given that DMD is characterized by muscular instability due to the absence of dystrophin, we employed “myomechanical profiling”—a custom apparatus compatible with an MCR702e rheometer—to evaluate stiffness, compressibility, and elasticity of the tibialis anterior muscle of male mice following a bout of submaximal isometric fatiguing exercise. Fatigue was standardized to a 50% reduction in strength. Immediately after exercise, both dystrophin‐positive (wildtype) and dystrophin‐deficient (*mdx*) muscles exhibited reduced compressibility. Storage and loss moduli, reflecting stiffness and energy dissipation during rotational deformation, increased markedly in fatigued wildtype muscle but remained unchanged in *mdx* muscle. Conversely, elasticity was unaffected in wildtype muscle but shifted *mdx* muscle toward a more viscous state. These findings indicate that compressibility, stiffness, and energy storage capacity are not disproportionately affected in dystrophin‐deficient muscle compared to wildtype muscle following fatiguing contractions. Thus, metabolically fatiguing, non‐lengthening contractions appear not to compromise viscoelastic properties in dystrophin‐deficient muscle, supporting their potential clinical use without exacerbating muscle instability.

## 1 INTRODUCTION

Duchenne muscular dystrophy (DMD) is an X‐linked neuromuscular disorder that affects hundreds of thousands of males worldwide (Crisafulli et al., 2020; Salari et al., 2022), with long‐term treatment options remaining uncertain (Reuters, 2025). Most patients become wheelchair‐dependent before adolescence, and without pharmaceutical, ventilatory, or physical therapy interventions, life expectancy rarely extends beyond the third decade. Muscle contraction—whether involuntary (e.g., cardiac) or voluntary (e.g., walking or exercise)—exacerbates disease progression because the muscle tissue is highly vulnerable to damage, degeneration, and loss of contractile function (Lindsay et al., 2020; Vohra et al., 2015). This vulnerability makes prescribing exercise for individuals with DMD particularly challenging to justify.

Muscle contractions can be categorized as isometric, isokinetic, concentric, or eccentric. Eccentric contractions—where the muscle lengthens under tension—are commonly used to evaluate therapeutic efficacy in DMD because skeletal muscle in this condition exhibits rapid strength loss and structural compromise (Head et al., 1992; Lindsay et al., 2020; Moens et al., 1993; Petrof et al., 1993). Traditional exercise, such as running, is generally contraindicated for DMD, as studies in mouse models indicate a narrow threshold between potential benefit and disease exacerbation (Hammer et al., 2021; Spaulding & Selsby, 2018). Conversely, both preclinical and clinical evidence suggest that isometric contractions—maintaining muscle length while under tension—while metabolically fatiguing, may offer therapeutic benefits. For instance, isometric resistance training in DMD mouse models has been shown to improve strength, increase satellite cell abundance, and enhance contractility (Lindsay, Larson, et al., 2019; Yamauchi et al., 2023, 2025). In patients with DMD, isometric resistance exercise (e.g., modified leg curls; knee extension and flexion) appears safe, tolerable, and capable of providing functional improvements (Lott et al., 2021) despite disruption to electromyography activity (Chen et al., 2025). However, the effects of eccentric and isometric exercise must be interpreted within the context of skeletal muscle stability, as DMD is fundamentally a disease characterized by compromised muscle integrity.

Muscle instability in DMD results from the loss of dystrophin function or expression (Hoffman et al., 1987). Dystrophin, a key component of the dystrophin–glycoprotein complex (DGC), provides a critical biomechanical link between the intracellular actin cytoskeleton and the extracellular matrix of muscle fibers (Rybakova et al., 2000). During exercise, dystrophin facilitates lateral force transmission from the sarcomere (Kumar et al., 2004), buffers mechanical stress through its shock‐absorbing properties (Le et al., 2018), and serves as a signaling hub for proteins involved in exercise‐related pathways, such as neuronal nitric oxide synthase (Lai et al., 2009). Consequently, assessing the impact of exercise on muscle stability and integrity—particularly biomechanical components—is essential for determining whether it is safe or exacerbates the underlying pathology of DMD.

Myomechanical profiling—an in vivo rheological technique that applies controlled compression and rotational shear transverse to muscle fiber orientation with millinewton precision and high spatial resolution—reveals that dystrophin‐deficient skeletal muscle in mice is stiffer and less elastic (Devananthan et al., 2025). Other methods have assessed passive and active mechanical properties in isolated myotubes (Pasternak et al., 1995), excised whole muscle (Lindsay, Southern, et al., 2019; Lopez et al., 2021), biopsies (Puttini et al., 2009), or entire muscle groups (Lindsay et al., 2021), applying force either along or across fiber planes. However, these data remain highly variable and difficult to interpret clinically for DMD. In the context of exercise, eccentric contractions induce severe strength loss in the anterior crural muscles of DMD mouse models and exacerbate underlying viscoelastic deficiencies measured via myomechanical profiling (Devananthan et al., 2025). This evidence strongly indicates that lengthening exercise under tension is detrimental to DMD muscle biomechanics, yet it remains unclear whether isometric exercise produces similar effects.

In dystrophin‐positive skeletal muscle, shear‐wave elastography typically shows that isometric training does not alter active or passive mechanical properties (Kubo et al., 2017), although a single bout of fatiguing isometric exercise reduces both passive and active stiffness (Morel et al., 2019; Nordez et al., 2009; Vincent et al., 2024). Under non‐exercise conditions, elastography reveals that patients with DMD exhibit increased muscle stiffness compared to resting dystrophin‐positive muscle (Pichiecchio et al., 2018), but this stiffness remains lower than that of healthy muscle in a contracted state (Bensamoun et al., 2015). However, the impact of fatiguing isometric exercise on skeletal muscle biomechanics in DMD remains unknown—particularly under transverse compressive loading combined with rotational strain in vivo for direct viscoelastic assessment. Given the clinical importance of understanding how non‐lengthening exercise influences muscle stability and biomechanics in DMD, this study employed myomechanical profiling to measure in vivo viscoelastic properties of dystrophin‐positive and dystrophin‐deficient skeletal muscle following a bout of fatiguing isometric exercise in mice. Results from transverse compression and rotational strain analysis in vivo indicate that isometric fatiguing exercise was well tolerated by dystrophin‐deficient skeletal muscle of mice and did not exacerbate the underlying stiffness or impaired elasticity characteristic of the disease.

## 2 METHODS

### 2.1 Ethical approval

All physiological and rheological studies were reviewed and approved by the University of Otago Animal Ethics Committee (AUP‐24‐15). All animals were housed in the Christchurch Animal Research Area (CARA) within the University of Otago, Christchurch (UOC) and treated in accordance with standards set by the University of Otago Animal Welfare Office.

### 2.2 Experimental mice

All animals were bred in‐house under breeding ethics reviewed and approved by the University of Otago Animal Ethics Committee (AUP‐23‐115). The mice were originally obtained from Jackson Laboratory, Maine, USA. Male C57BL/10ScSn‐J (WT) and C57BL/10ScSn‐*mdx* (*mdx*) mice between 9 and 11 weeks of age were used for studies (WT = 7; 10.7 ± 0.2 weeks of age, *mdx* = 6; 10.3 ± 0.4 weeks of age). All mice were housed in groups of three to four per cage on a 12/12 h light/dark cycle with food (Biological Associates Australia; Teklad 2918) and water provided ad libitum.

### 2.3 Experimental design

To determine the impact of isometric fatiguing exercise on the viscoelastic properties of dystrophin‐positive and dystrophin‐deficient skeletal muscle, WT and *mdx* mice were anesthetized, and then they completed a bout of submaximal isometric fatiguing exercise of the anterior crural muscles (tibialis anterior, extensor digitorum longus, and extensor hallucis longus). After the exercise protocol, while remaining under anesthesia, the exercised (left leg; fatigued) and contralateral non‐exercised (right leg; non‐fatigued; control) tibialis anterior muscles of WT and *mdx* mice were myomechanically profiled to assess stiffness and elasticity under transverse compression and rotational strain. Immediately after myomechanical profiling was completed on a mouse, it was euthanized by cervical dislocation while under anesthesia.

### 2.4 Methods

#### 2.4.1 Anesthesia

Mice were initially anesthetized in an induction chamber using 5% isoflurane and then maintained by the inhalation of 1%–3% isoflurane mixed with oxygen at a flow rate of 100 mL·min^−1^.

#### 2.4.2 Muscle physiology

Maximal isometric torque of the anterior crural muscles was measured as previously described (Devananthan et al., 2025; Lindsay et al., 2020). The anesthetized mouse was placed on a temperature‐controlled platform to maintain core temperature at 37°C. The left knee was clamped, and the left foot was secured to an aluminium footplate attached to the shaft of the servo motor system (Model 300B‐LR; Aurora Scientific, Aurora, Ontario, Canada). Sterilized platinum needle electrodes percutaneously stimulated the left common peroneal nerve, which was connected to the stimulator and stimulus isolation unit. The contractile function of the anterior crural muscles (including the tibialis anterior) was assessed by measuring isometric torque (150 Hz; 200 ms train with 0.1‐ms pulses) every 45 s until peak torque was achieved (within 0.1 mN.m). One minute later, the anterior crural muscles performed a torque frequency analysis (10, 20, 30, 40, 50, 60, 80, 100, 150, 200 Hz) with 45 s between contractions. The frequency required to generate 50% of peak torque (Figure S1 Supplementary material) was used to perform submaximal isometric fatiguing contractions. Two minutes after the torque frequency, mice completed a series of submaximal isometric fatiguing contractions with 10 s between contractions, continuing until 50% torque loss or 150 contractions (whichever occurred first). The number of contractions to lose submaximal isometric torque was recorded, and then the anterior crural muscles were exposed to a single 150 Hz 200 ms train to determine peak torque lost. The mouse was then transferred under anesthesia to myomechanical profiling as described below.

#### 2.4.3 Myomechanical profiling

Myomechanical profiling (Devananthan et al., 2025) enables the in vivo assessment of the viscoelastic properties of murine skeletal muscle with high measurement sensitivity and spatial resolution, and allows for application of compression and rotational shear transverse to fiber orientation. Storage and loss moduli, as functions of shear strain, display the responses to compressive force and rotational shear. Stiffness, energy storage and dissipation, and elasticity of muscle can be assessed in response to an intervention or to differentiate healthy from diseased muscle.

The tibialis anterior muscle of anesthetized mice was exposed and isolated under a microscope from the fascia, extensor digitorum longus muscle, and tibia–this approach enabled an isolated myomechanical profiling characterization of the tibialis anterior without interference from surrounding musculature, tendon, or bone as previously described (Devananthan et al., 2025).

After isolation of the tibialis anterior, the mouse was transferred under anesthesia to our myomechanical profiling rig attached to an MCR702e MultiDrive Dynamic Material Analyzer rheometer (Anton Paar). The mouse in the supine position, with the fitted anesthesia nose cone, was supported on a flat platform maintained at 37 ± 2°C. The temperature was monitored using an infrared thermometer. A rigid 80 × 4 × 0.5 mm stainless steel plank was inserted between the tibialis anterior muscle and the extensor digitorum longus muscle/tibia, placed into the slots in the rest holder posts, and securely clamped in position on both ends. The platform height was adjusted to achieve a 90‐degree angle between the femur and tibia. The knee was clamped to ensure the tibialis anterior aligned centrally with the probe. The foot was immobilized and taped to a footrest. The apparatus interfaced the rheometer so that the exposed muscle was located directly under a custom 3 mm cylindrical rheometer probe, with the plank aligned centrally with the probe.

The tibialis anterior muscle first underwent pre‐compression of 0.01 N at 10 μm/s (indicating full contact of the probe with the muscle surface and an assessment of muscle thickness). Then, the muscle was further compressed by 20% of its original thickness at 10 μm/s–a change in thickness that differentiates the viscoelastic properties of dystrophin‐positive and dystrophin‐deficient skeletal muscle while maintaining secure contact and causing no damage to the underlying architecture (Devananthan et al., 2025). One minute later, while maintaining the compressive force to achieve a 20% loss in thickness, a rotational shear sweep over a range of ascending shear strain amplitudes was completed, starting at a shear strain of 0.001% and ramping logarithmically to 10%. The shear strain within the muscle was induced by the probe's oscillatory rotation relative to its original position at 1 Hz. The muscle resistance to this deformation was measured via the storage and loss moduli.

### 2.5 Statistics

Data were analyzed in GraphPad Prism 10.0. Data were assessed for normality by the Shapiro–Wilk normality test. Genotype comparisons for a single measure (e.g., muscle thickness) were assessed using an unpaired *t*‐test. For all other data, and if normally distributed, a two‐way non‐repeated or repeated measures ANOVA or non‐parametric equivalent (e.g., Friedman's test) were used with Bonferroni post‐hoc analysis. All data are presented as mean ± SEM and *p* < 0.05 was required for significance.

## 3 RESULTS

### 3.1 Physiology

**FIGURE 1:**
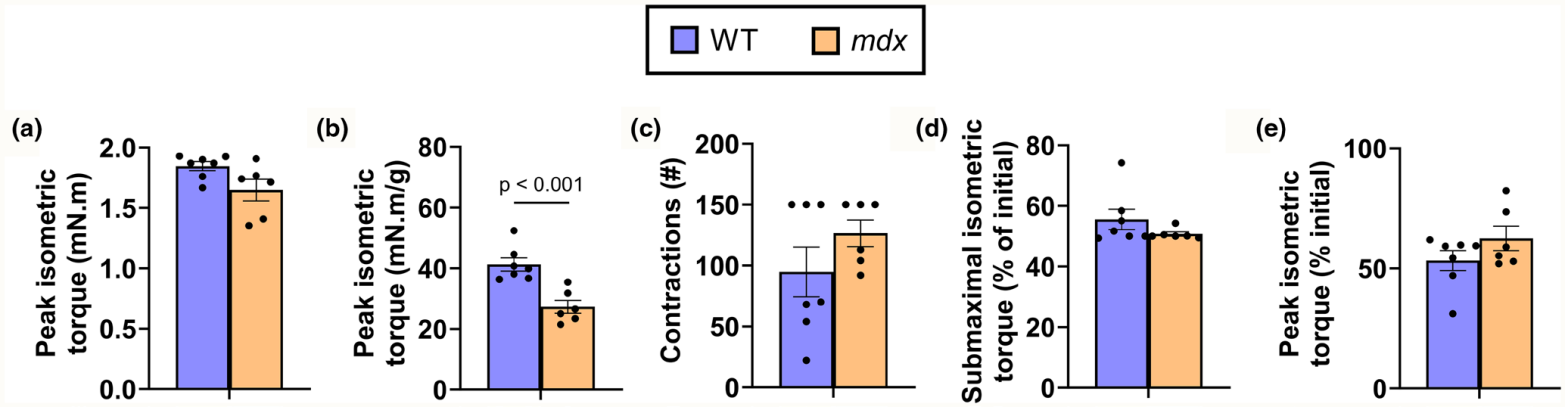
*Mdx* skeletal muscle is weaker but as resilient to submaximal isometric fatiguing exercise as wildtype (WT) skeletal muscle. (a) Absolute strength and (b) relative strength of the anterior crural muscles (tibialis anterior, extensor digitorum longus, and extensor hallucis longus) of dystrophin‐positive (WT) and dystrophin‐deficient (*mdx*) mice. (c) Total number of submaximal isometric fatiguing contractions (d) to lose 50% torque or to achieve 150 total contractions. (e) Peak isometric torque of the anterior crural muscles immediately following the series of submaximal isometric fatiguing contractions. Data are mean ± SEM. *N* = 7 and 6 for WT and *mdx* mice, respectively. *p*‐values <0.05 are shown.

Absolute strength of the anterior crural muscles was not different between WT and *mdx* mice (Figure 1a; *p* = 0.052), but *mdx* anterior crural muscles were relatively weaker when normalized to tibialis anterior mass (Figure 1b; *p* < 0.001).

The total number of submaximal isometric fatiguing contractions to lose 50% torque or reach 150 total contractions was not different between WT and *mdx* mice (Figure 1c; *p* = 0.219). Submaximal isometric fatiguing contractions resulted in a loss of 45.5% and 46.7% submaximal and peak torque, respectively, for WT mice and 49.3% and 37.5% submaximal and peak torque, respectively, for *mdx* mice (Figure 1d,e; *p* = 0.184).

### 3.2 Static compression

**FIGURE 2:**
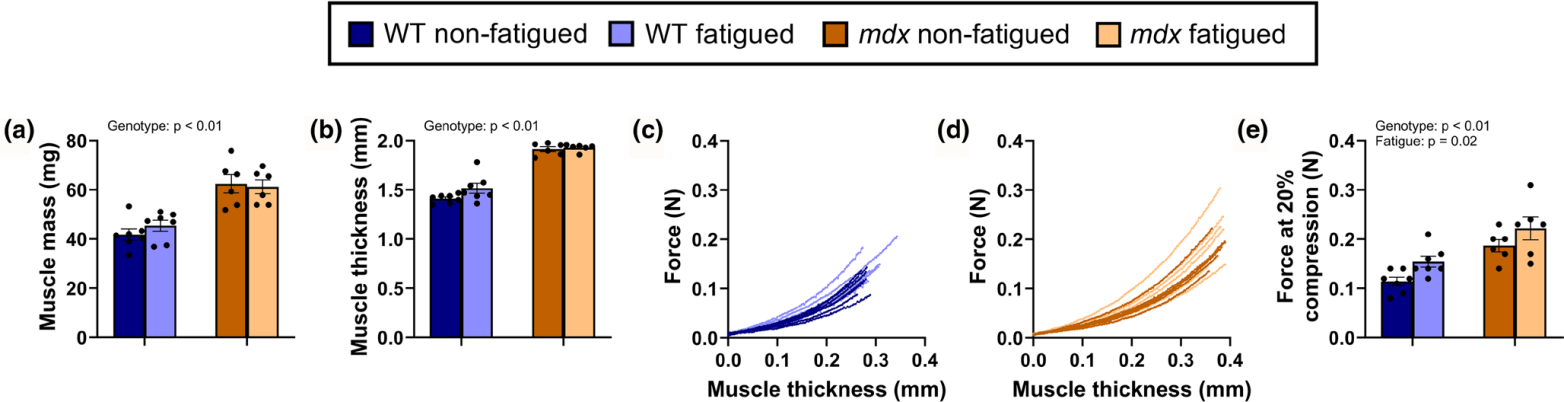
Fatiguing exercise increases transverse skeletal muscle stiffness. (a) Non‐fatigued (control) and fatigued tibialis anterior muscle mass and (b) muscle thickness of wildtype (WT) and *mdx* mice. (c) Dynamic changes in compressive force required to compress non‐fatigued (control) and fatigued tibialis anterior muscle of WT and (d) *mdx* mice to 80% of original thickness (20% loss). (e) Force required to compress the non‐fatigued and fatigued tibialis anterior of WT and *mdx* mice to 80% of original thickness. Data are mean ± SEM. *N* = 7 and 6 for WT and *mdx* mice, respectively. Only terms with *p*‐values <0.05 are shown.

The tibialis anterior muscles of *mdx* mice were heavier (Figure 2a; *p* < 0.001) and thicker (Figure 2b; *p* < 0.001) relative to WT mice. A 50% loss in submaximal isometric torque of the anterior crural muscles did not affect the mass or the thickness of the tibialis anterior of WT or *mdx* mice (*p* ≥ 0.079). During myomechanical profiling, compression to achieve a 20% reduction in tibialis anterior thickness in non‐fatigued and fatigued muscle (Figure 2,c,d) required greater force in *mdx* mice relative to WT mice (Figure 2e; *p* < 0.001). The fatigued tibialis anterior of WT and *mdx* mice also required greater force to compress by 20% of original thickness relative to the non‐fatigued tibialis anterior muscle (*p* = 0.016), but there was no difference in the percent increase in force required to compress a fatigued tibialis anterior in WT mice relative to *mdx* mice (41.4 ± 16.8% vs. 18.7 ± 9.5%; *p* = 0.288). These data indicate that submaximal isometric fatiguing contractions transversely stiffen skeletal muscle, but that dystrophin‐deficiency does not further affect this exercise‐induced effect.

### 3.3 Rotational shear

**FIGURE 3:**
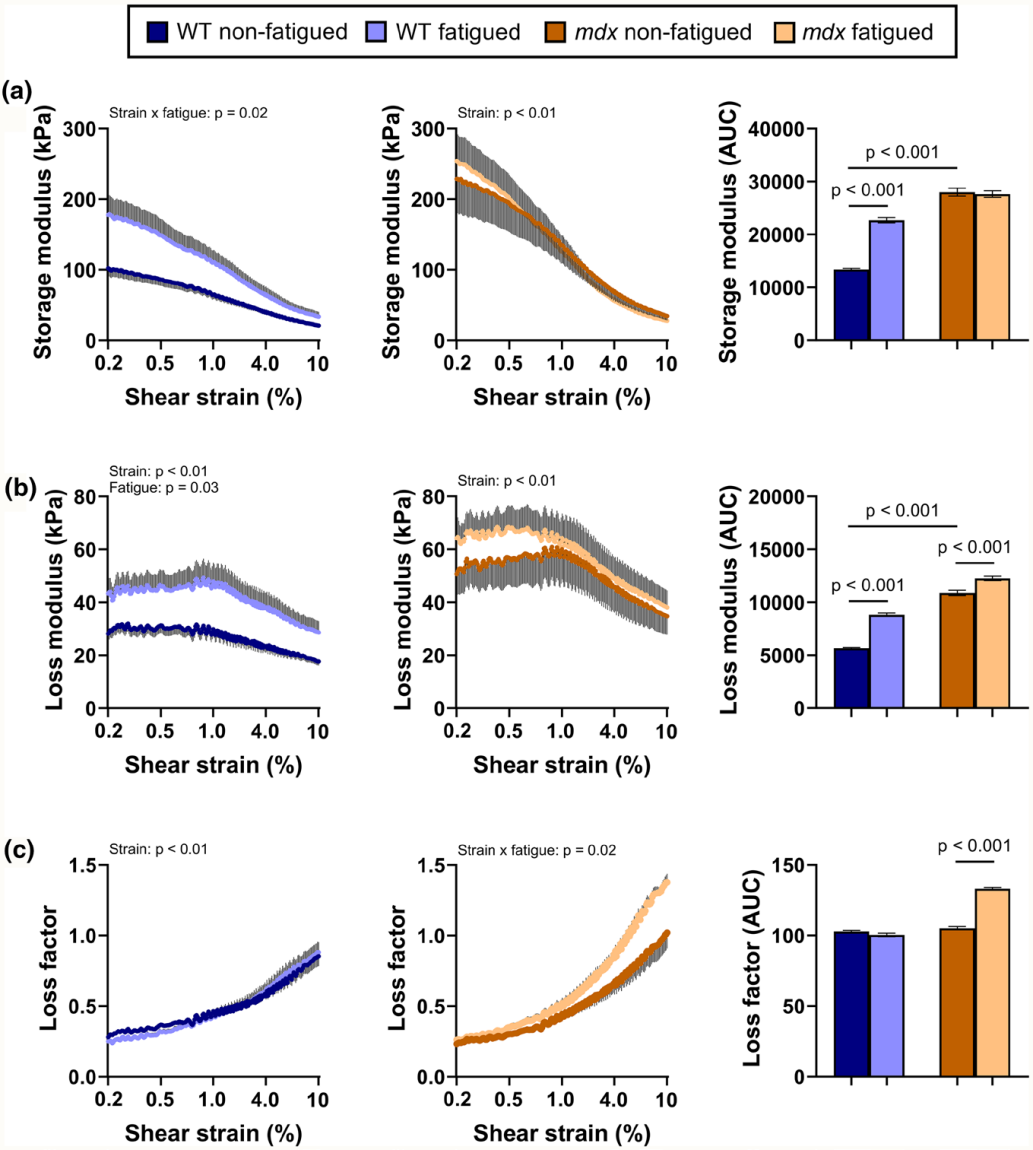
Dystrophin‐deficient skeletal muscle is resistant to fatiguing exercise‐induced changes in stiffness but loses its elasticity. (a) Storage modulus, (b) loss modulus, (c) loss factor and associated areas under the curve (AUC) for wildtype (WT) and *mdx* non‐fatigued and fatigued tibialis anterior skeletal muscle. Data are mean ± SEM. *N* = 7 and 6 for WT and *mdx* mice, respectively. Only terms and interactions with *p*‐values <0.05 are shown.

Dystrophin‐deficient tibialis anterior muscle of *mdx* mice has a larger storage modulus at low rotational shear strain relative to WT tibialis anterior muscle (*p* < 0.001). Submaximal isometric fatiguing contractions increased the storage modulus of the tibialis anterior muscle of WT mice but not *mdx* mice (Figure 3a). There was a shear strain × fatigue interaction for storage modulus for WT mice (*p* = 0.016), where the fatigued tibialis anterior was on average 1.7‐fold stiffer than the non‐fatigued tibialis anterior across shear strains of 0.001%–10%. In contrast, submaximal isometric fatiguing contractions did not affect the storage modulus of the tibialis anterior of *mdx* mice (*p* = 0.961). Storage modulus of tibialis anterior muscle in WT and *mdx* mice decreased with increasing shear strains, and while there was no effect of submaximal isometric fatiguing contractions on these properties for WT mice (*p* = 0.591), there was a strain × fatigue interaction for *mdx* mice (*p* = 0.042)–storage modulus of *mdx* tibialis anterior decreases faster with increasing rotational strain. These data indicate that although dystrophin deficiency stiffens skeletal muscle of mice in response to transverse compression and rotational shear, it is resistant to exacerbation of this biomechanical phenotype by submaximal isometric fatiguing contractions.

Dystrophin‐deficient tibialis anterior muscle of *mdx* mice dissipates more energy during rotational strain (i.e., higher loss modulus) relative to WT tibialis anterior muscle (*p* < 0.001). Submaximal isometric fatiguing contractions increased the loss modulus of the tibialis anterior muscle of WT mice but not *mdx* mice (Figure 3b). Shear strain (*p* < 0.001) and fatigue (*p* = 0.035) increased the loss modulus for WT mice, where the fatigued tibialis anterior dissipated on average 1.6‐fold more energy than the non‐fatigued tibialis anterior across shear strains of 0.001%–10%. In contrast, submaximal isometric fatiguing contractions did not affect the loss modulus of the tibialis anterior of *mdx* mice when assessed across all shear strains (*p* = 0.610), but an area under the curve analysis indicated an increase in energy dissipation overall (*p* < 0.001). Tibialis anterior muscle of WT and *mdx* mice dissipated more energy at lower rotational shear strain (0.001%–1%) relative to higher rotational shear strain (1%–10%; *p* < 0.001), and this effect was maintained for WT tibialis anterior in response to submaximal isometric fatiguing contractions but not for *mdx* tibialis anterior. These data indicate that although dystrophin‐deficiency increased energy dissipation of skeletal muscle of mice in response to transverse loading with additional shear, it is not as susceptible as WT skeletal muscle to increased energy dissipation properties when exposed to submaximal isometric fatiguing contractions.

The loss factor quantifies the relationship between storage and loss moduli, with ratios greater than one indicating a material, in this case, skeletal muscle, is more liquid‐like (i.e., more viscous than elastic) (Zemła et al., 2021). Across the shear strain amplitudes, both WT and *mdx* tibialis anterior skeletal muscle had a loss factor less than one, and there was no difference between genotypes (Figure 3c; *p* = 0.488). However, when exposed to submaximal isometric fatiguing contractions, *mdx* tibialis anterior became more liquid‐like at high rotational shear (1.36 at 10%; *p* < 0.001), but WT tibialis anterior was unaffected (*p* = 0.380). These data indicate that submaximal isometric fatiguing contractions reduced the elastic component of dystrophin‐deficient skeletal muscle.

## 4 DISCUSSION

This study aimed to evaluate the effects of submaximal isometric fatiguing exercise on the viscoelastic properties of dystrophin‐deficient skeletal muscle using myomechanical profiling. The collective profile of *mdx* muscle demonstrated resilience to viscoelastic alterations following submaximal isometric fatigue, with changes in stiffness, energy dissipation, elasticity, and viscosity comparable to those observed in dystrophin‐positive muscle. These findings suggest that a single bout of submaximal isometric contractions does not adversely impact the biomechanical properties of DMD‐like skeletal muscle and may not exacerbate the pathological biomechanical phenotype.

### 4.1 Biomechanics of dystrophin‐deficiency in skeletal muscle

Myomechanical profiling and physiological analyses revealed that dystrophin deficiency in C57BL/10 *mdx* mice weakens skeletal muscle, increases muscle thickness, and stiffens tissue under static compression without reducing resilience to submaximal isometric contractions. These results align with many previous studies (Lindsay, Southern, et al., 2019; Petrof et al., 1993; Sharp et al., 2011; Trost et al., 2021; Yang et al., 2012); however, the observed resistance to fatigue contrasts with findings from other studies using muscles with similar fiber‐type composition (Wineinger et al., 1998). Although *mdx* skeletal muscle exhibits numerous metabolic defects (Freidenberg & Olefsky, 1985; Lindsay et al., 2018; Rybalka et al., 2014), this outcome was unexpected. It is possible that the individualized, submaximal isometric fatiguing protocols minimized variability, suggesting that under comparable exercise conditions, dystrophin deficiency preserves resistance to metabolically induced strength loss. The combination of a 51% reduction in relative muscle strength and a 64% increase in static stiffness under transverse loading provides further evidence that the absence of dystrophin negatively impacts skeletal muscle quality, function, and structural stability.

### 4.2 Biomechanical impact of fatiguing exercise

In dystrophin‐positive skeletal muscle, submaximal isometric fatiguing exercise typically reduces stiffness (Morel et al., 2019; Nordez et al., 2009; Vincent et al., 2024). Conversely, under non‐exercised conditions, dystrophin‐deficient muscle is generally stiffer and less elastic (Devananthan et al., 2025; Lacourpaille et al., 2015; Lindsay, Southern, et al., 2019; Lopez et al., 2021). Our findings show that when WT and *mdx* skeletal muscles were subjected to a bout of submaximal isometric fatiguing exercise—each losing 50% of initial strength—both exhibited increased transverse stiffness under static compressive loading. To standardize compression, we individualized the applied load so that each muscle was compressed by 20% of its original thickness. Without this approach, WT muscle would have experienced less compression under the same load, potentially biasing subsequent rotational shear analyses. Notably, dystrophin‐deficient *mdx* skeletal muscle was no more stiffened than WT muscle following exercise, suggesting that fatiguing contractions do not exacerbate transverse stiffness in the absence of dystrophin.

Across the rotational shear spectrum (0.001%–10%), storage modulus in skeletal muscle is highest at low rotational strains, independent of genotype, suggesting that increased fiber recruitment during larger rotations reduces storage modulus (see figure 11 in Devananthan et al. (2025)). Following a bout of fatiguing exercise under individualized constant compression, *mdx* skeletal muscle showed complete resilience to changes in storage modulus during rotational shear. In contrast, WT muscle exhibited an almost 100% increase in storage modulus following submaximal isometric fatiguing contractions. These findings indicate that although dystrophin deficiency stiffens skeletal muscle under transverse compression with additional shear under non‐exercised conditions, it does not exacerbate this biomechanical phenotype following a bout of fatiguing exercise. These results suggest that this type of isometric fatiguing exercise is biomechanically safe and tolerable in the *mdx* mouse model of DMD.

Static compression and rotational shear had minimal impact on *mdx* skeletal muscle following isometric fatiguing exercise, supporting the potential therapeutic role of exercise modality for DMD. Complementing these stiffness‐related findings, analysis of the loss modulus revealed that although dystrophin‐deficient skeletal muscle dissipates more energy than it stores across the rotational shear spectrum, it does not exhibit increased energy dissipation following fatiguing exercise, despite comparable strength loss to WT skeletal muscle. This contrasts sharply with the substantial increase in loss factor observed in *mdx* skeletal muscle after a bout of submaximal isometric fatiguing contractions. This outcome suggests a possible adverse effect of submaximal isometric fatiguing exercise as the dystrophin‐deficient muscle shifts toward a more viscous state, as indicated by changes in the loss factor measurement. The loss factor, a unitless measure of tissue behavior under compression and shear (<1 = elastic; >1 = viscous), remained well below one after eccentric contractions (Devananthan et al., 2025), suggesting preserved elasticity despite severe damage. In contrast, the data in this study indicate that a bout of submaximal isometric fatiguing exercise reduces the elastic component of *mdx* muscle, but not WT muscle, implying that this exercise modality may impair structural stability in dystrophin‐deficient muscle.

### 4.3 Physiology to myomechanical platform

Myomechanical profiling, which enables millinewton precision in assessing skeletal muscle biomechanics under transverse loading and rotational shear in anesthetized mice, was integrated with in vivo physiological measurements to evaluate the impact of exercise on viscoelastic properties. This physiology‐to‐myomechanical pipeline allows researchers to maintain anesthesia throughout the intervention and immediately assess biomechanical changes following physiological challenges (e.g., fatigue or injury). The setup also permits the non‐intervened muscle to serve as an intra‐mouse control, and the entire process can be completed within 1 h per animal. This unique in vivo approach provides timely, precise, and repeatable evaluation of how physiological interventions influence skeletal muscle biomechanics under basal conditions, disease states, or following therapeutic interventions.

## 5 LIMITATIONS

This study isolated the tibialis anterior of mice to understand the impact of submaximal isometric fatiguing contractions on the viscoelastic properties–thus, the limitations of this technology are identical to that of our previous study (Devananthan et al., 2025). There were no additional limitations to this study.

## 6 CONCLUSION

Leveraging the in vivo precision of myomechanical profiling, we determined that a single bout of submaximal isometric fatiguing contractions does not exacerbate compressive or rotational stiffness in dystrophin‐deficient skeletal muscle, although it may impair the elastic behavior of muscle. Overall, the absence of dystrophin does not appear to negatively influence skeletal muscle's response to metabolically induced strength loss. These findings suggest that prescribing this form of exercise could be clinically beneficial for DMD, given the metabolic adaptations normally associated with non‐injurious muscle contractions.

## AUTHOR CONTRIBUTIONS

**Deirdre L. Merry:** Data curation; formal analysis; investigation; methodology. **Pavithran Devananthan:** Data curation; investigation; methodology. **Natalia Kabaliuk:** Data curation; funding acquisition; methodology; resources; supervision. **Angus Lindsay:** Conceptualization; data curation; formal analysis; funding acquisition; investigation; methodology; project administration; resources; software; supervision.

## FUNDING INFORMATION

The Health Research Council (Sir Charles Hercus Health Research Fellowship [23/037], Angus Lindsay) and University of Canterbury Biomolecular Interaction Centre (Seed Funding, Natalia Kabaliuk) supported this work. The funding agencies had no input in study design; in the collection, analysis, and interpretation of data; in the writing of the report; and in the decision to submit the article for publication.

## CONFLICT OF INTEREST STATEMENT

The authors have no competing interests to declare.

## Supporting information


Figure S1

## Data Availability

Data will be made available at the reasonable request of the corresponding author.
