# Supplementary material for: Viscoelastic properties of dystrophin‐deficient mouse skeletal muscles are resilient to isometric fatiguing exercise
Source: Physiol Rep. 2026 Mar 19;14(6):e70841. doi: 10.14814/phy2.70841 (PMC13098110; doi:10.14814/phy2.70841)
Supplement: Supplementary file 1 — Figure S1 [file PHY2-14-e70841-s001.pdf]

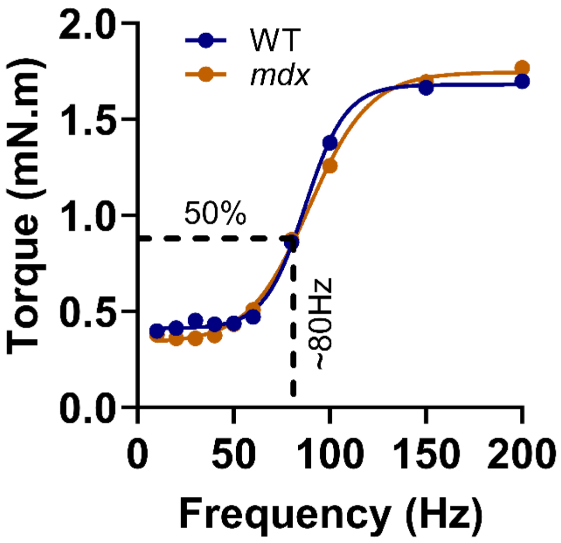

Figure S1. Representative *in vivo* torque frequency curves of wildtype (WT) and *mdx* mouse anterior crural muscles highlighting the frequency used to generate 50% of max torque for the submaximal isometric fatiguing contractions.
